# Extracorporeal shockwave therapy as a key component of interdisciplinary wound management in diabetic foot syndrome: A case report

**DOI:** 10.1007/s10354-026-01184-1

**Published:** 2026-08-17

**Authors:** Richard Crevenna, Zoltan Vass, Michael Mickel, Michael Leutner, Shuren Li, Mohammad Keilani

**Affiliations:** 1 Department of Physical Medicine, Rehabilitation and Occupational Medicine, Medical University of Vienna, University Hospital General Hospital Vienna, Vienna Healthcare Group, Vienna, Austria; 2 Comprehensive Center for Musculoskeletal Disorders (CCMSD), Medical University of Vienna, University Hospital General Hospital Vienna, Vienna Healthcare Group, Vienna, Austria; 3 Competence Center for Occupational Safety and Health Promotion, University Hospital General Hospital Vienna, Vienna Healthcare Group, Vienna, Austria; 4 Department of Internal Medicine I, Infectiology, Medical University of Vienna, University Hospital General Hospital Vienna, Vienna Healthcare Group, Vienna, Austria; 5 Department of Internal Medicine III, Endocrinology and Metabolism, Medical University of Vienna, University Hospital General Hospital Vienna, Vienna Healthcare Group, Vienna, Austria; 6 Department of Radiology and Nuclear Medicine, Clinical Division of Endocrinology & Metabolism, Medical University of Vienna, University Hospital General Hospital Vienna, Vienna Healthcare Group, Vienna, Austria

**Keywords:** Wounds, Diabetic foot syndrome, Case presentation, Mechanotransduction, Adjunctive regenerative physical therapy, Wunden, Diabetisches Fußsyndrom, Fallpräsentation, Mechanotransduktion, Additive physikalische Therapie

## Abstract

**Background:**

A 57-year-old male patient presented with the first manifestation of severe type 2 diabetes mellitus in the setting of diabetic foot syndrome complicated by osteomyelitis.

**Methods:**

The patient was treated conservatively within a multimodal interdisciplinary approach. In addition, focused extracorporeal shockwave therapy (fESWT) was performed according to International Society for Medical Shockwave Treatment (ISMST)-oriented principles.

**Results:**

After five sessions of fESWT, local wound management resulted in extraction of a relevant bony sequestrum. Following sequestrum removal, a large wound cavity remained in the region of the right first toe (digitus I pedis), corresponding approximately to the size of an entire phalanx. Remarkably, within only 4 days, rapid and substantial granulation of the deep wound cavity was observed.

**Conclusion:**

The present case supports the hypothesis that focused ESWT may represent a clinically relevant adjunctive treatment option in selected patients with diabetic foot syndrome and complicated wound healing disorders. At the same time, it highlights the need for prospective randomized controlled trials.

## Abstract (de)

**Hintergrund:**

Ein 57-jähriger männlicher Patient mit einer Erstmanifestation eines ausgeprägten Diabetes mellitus Typ 2 stellte sich im Rahmen eines diabetischen Fußsyndroms mit komplizierender Osteomyelitis als Erstmanifestation zur Behandlung vor.

**Methoden:**

Der Patient wurde konservativ in Form einer diabetologischen Einstellung sowie eines multimodalen, interdisziplinären Wundmanagements behandelt. Zusätzlich wurde eine fokussierte extrakorporale Stoßwellentherapie (fESWT) gemäß der aktuellen Leitlinie der International Society for Medical Shockwave Treatment (ISMST) durchgeführt.

**Ergebnisse:**

Nach 5 Sitzungen fESWT führte die lokale Wundversorgung zur Abstoßung eines relevanten Knochensequesters. Danach verblieb im Bereich der rechten großen Zehe (Digitus I pedis) eine große Wundhöhle (etwa so groß wie eine Phalanx). Innerhalb von nur 4 Tagen wurde eine rasche und fast vollständige Granulation der tiefen Wundhöhle beobachtet.

**Schlussfolgerung:**

Die Kasuistik stützt die Hypothese, dass die fokussierte ESWT bei ausgewählten Patienten mit diabetischem Fußsyndrom und komplizierten Wundheilungsstörungen eine klinisch relevante ergänzende Behandlungsoption darstellen kann. Gleichzeitig unterstreicht dieser Fallbericht die Notwendigkeit prospektiver randomisierter kontrollierter Studien.

## Background

Diabetic foot syndrome (DFS) represents one of the most clinically and socioeconomically relevant complications of diabetes mellitus and remains associated with substantial morbidity, impaired quality of life, and high healthcare costs [1, 2]. Chronic ulceration, infection, osteomyelitis, pain, mobility impairment, and the risk of minor or major amputation frequently result in severe limitations to function, activity, and participation, as defined by the International Classification of Functioning, Disability, and Health (ICF) [3]. Consequently, contemporary DFS management requires an interdisciplinary and multimodal treatment strategy including infection control, pressure off-loading, stage-adapted wound care, metabolic optimization, revascularization strategies where appropriate, and physical medicine interventions [3, 4].

Extracorporeal shockwave therapy (ESWT), particularly focused ESWT (fESWT), has increasingly gained attention as an adjunctive regenerative physical therapy in chronic diabetic foot ulcers [4–6]. Meta-analyses and clinical studies suggest beneficial effects on wound healing, tissue oxygenation, microcirculation, and tissue regeneration in diabetic foot ulcers [3, 7, 8]. Extracorporeal shockwave therapy is primarily considered an additive treatment modality in chronic, treatment-resistant, and poorly healing wounds in combination with established standard care [8, 9].

Proposed biological mechanisms of ESWT include stimulation of angiogenesis, improvement of microcirculation and tissue oxygenation, anti-inflammatory effects, release of growth factors, activation of cellular repair pathways, and promotion of regenerative and reparative tissue processes. Furthermore, several molecular signaling pathways have been implicated, including VEGF-, PI3K/Akt‑, ERK/MAPK-, and TGF-β-mediated mechanisms [10, 11].

Against this backdrop, we report a patient with diabetic foot syndrome and osteomyelitis in whom pronounced granulation of a deep wound cavity was observed within only a few days following focused ESWT integrated into a multimodal conservative treatment concept after several weeks of wound stagnation.

## Case presentation

A 57-year-old male patient presented with the first manifestation of severe type 2 diabetes mellitus in the setting of diabetic foot syndrome complicated by osteomyelitis. Apart from diabetes-associated findings, no relevant comorbidities, previous chronic diseases, or clinically significant laboratory abnormalities were identified.

The patient was treated conservatively within a multimodal interdisciplinary treatment concept including structured wound care, systemic antibiotic therapy, metabolic stabilization, and initiation of pharmacological and dietary diabetes management. The hemoglobin A1c level improved from pathological to normal values. In addition, focused extracorporeal shockwave therapy (fESWT) was performed according to ISMST-oriented treatment principles [12, 13] using a PiezoWave^2^ device (Richard Wolf GmbH, Germany).

Focused ESWT was applied according to ISMST-oriented protocols for chronic wounds using low- to medium-energy settings (approximately 0.08–0.25 mJ/mm^2^), 1000–2000 impulses per session, at a frequency of 3–5 Hz, usually once weekly. Treatment was performed peri-ulcerously without local anesthesia as part of a multimodal wound management concept [12, 13].

**Fig. 1:**
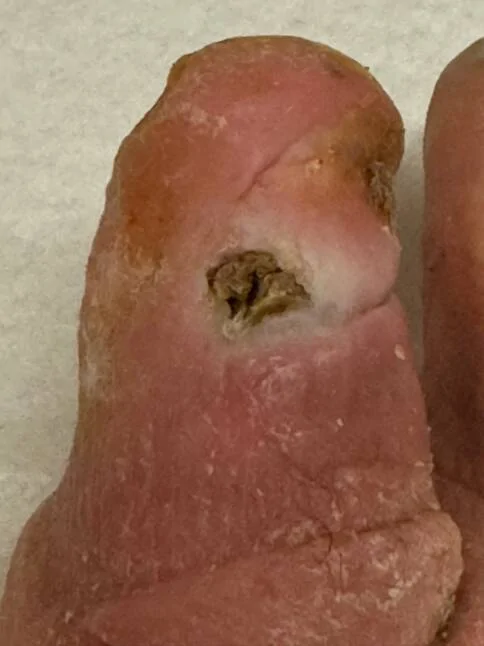
After five sessions of focused extracorporeal shockwave therapy (fESWT), local wound management resulted in extraction of a relevant bony sequestrum

**Fig. 2:**
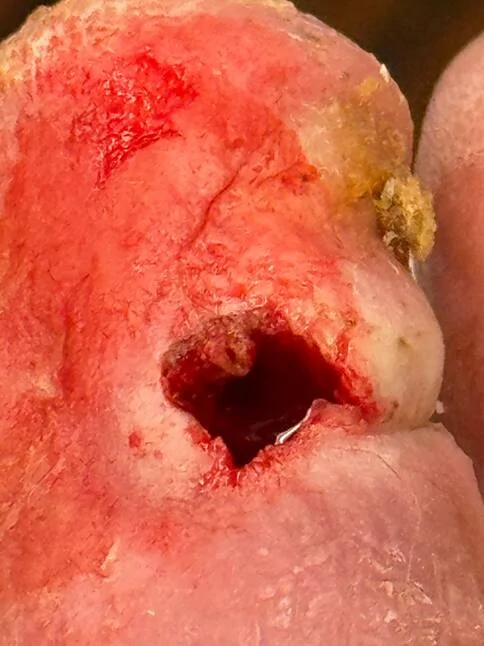
Following sequestrum removal, a large wound cavity remained in the region of the right first toe (digitus I pedis), corresponding approximately to the size of an entire phalanx

**Fig. 3:**
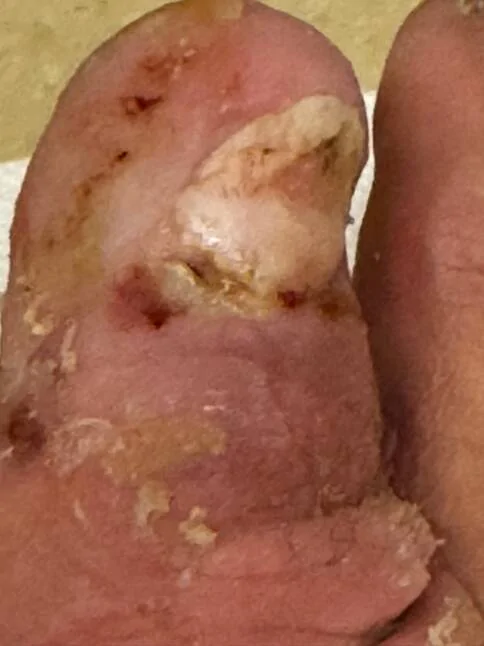
Remarkably, within only 4 days, rapid and substantial granulation of the deep wound cavity was observed

After five sessions of fESWT, local wound management resulted in extraction of a relevant bony sequestrum. Following sequestrum removal, a large wound cavity remained in the region of the right first toe (digitus I pedis), corresponding approximately to the size of an entire phalanx. Remarkably, within only 4 days, rapid and substantial granulation of the deep wound cavity was observed (Figs. 1, 2 and 3).

Subsequently, conservative treatment including wound care, pressure off-loading, infection control, metabolic optimization, and close clinical follow-up was continued.

## Discussion and conclusion

The present case illustrates the potential regenerative and reparative effects of focused extracorporeal shockwave therapy within a multimodal treatment approach for diabetic foot syndrome complicated by osteomyelitis. Particularly remarkable was the clinical dynamic observed in this patient: after approximately 5 weeks of largely stagnant wound healing, marked granulation of the wound cavity occurred within only 4 days following fESWT-associated treatment.

Although causal conclusions cannot be derived from a single case report, the observed temporal association appears clinically highly interesting, especially in view of the biological mechanisms attributed to ESWT [4, 6, 11]. Proposed effects include improvement of microcirculation and tissue oxygenation, angiogenic stimulation, modulation of inflammatory activity, and activation of regenerative cellular pathways [4, 6, 9, 11]. These mechanisms may be particularly relevant in chronic diabetic foot wounds characterized by impaired perfusion and prolonged healing processes.

The present case further underscores the importance of regenerative physical modalities as an integral component of modern DFS management. Clearly, ESWT should not be regarded as a replacement for established wound care strategies but rather as an adjunctive modality embedded within a structured multimodal treatment concept including infection management, wound care, off-loading, metabolic control, and interdisciplinary follow-up.

Particularly in patients with chronic and treatment-resistant wounds, fESWT may potentially function as a biological “therapeutic enabler” by stimulating regenerative processes and thereby improving the prerequisites for subsequent healing. This appears clinically relevant because successful wound healing in DFS extends far beyond local tissue closure and may contribute to pain reduction, preservation of mobility and ambulation, improvement of function and participation, and potentially reduction of the amputation risk.

The present case therefore supports the hypothesis that focused ESWT may represent a clinically relevant adjunctive treatment option in selected patients with diabetic foot syndrome and complicated wound healing disorders. At the same time, it highlights the need for prospective controlled studies to further define patient selection, treatment protocols, technical parameters, and long-term clinical outcomes.

## Data Availability

All data supporting the findings of this work are included in the article.
